# Life Events and Loneliness Transitions Among Middle-Aged and Older Adults Around the World

**DOI:** 10.1093/geroni/igaf122.426

**Published:** 2025-12-31

**Authors:** Mara Sheftel, Rachel Margolis, Ashton Verdery

**Affiliations:** Rutgers University, New Brunswick, New Jersey, United States; Western University, London, Ontario, Canada; The Pennsylvania State University, University Park, Pennsylvania, United States

## Abstract

**Objectives:**

Adult loneliness is a substantial social problem and a growing point of concern for policymakers around the world. We assess whether the predictors of loneliness onset among middle-aged and older adults vary from country to country in a large array of settings across world regions. Taking a life course perspective, we focus on common life events in our focal age range, including changes in partnership, coresidence, work, and health, and we test whether changes in them have comparable prospective associations with loneliness onset in different countries.

**Methods:**

We draw on respondent-level data from a diversity of world regions surveyed in 7 harmonized cross-national studies in 20 countries, representing 47% of the global population over the age of 50. Our innovative longitudinal approach estimates prospective transition probability models that examine how each life event predicts the transition into loneliness.

**Results:**

Despite substantial variation in the prevalence of loneliness and life events across the range of countries in our sample, our results highlight consistency in the predictors of loneliness transitions. Family and household changes like divorce, coresidence, and especially widowhood are paramount predictors of loneliness transition across settings, with changes in work and health playing more minor and less universal roles.

**Discussion:**

The results demonstrate the importance that family and household connections play in determining loneliness at these ages. These findings suggest that addressing late-life loneliness may require a focus on key life events, especially those concerning changes in families and households.

